# Biological nitrogen fixation in theory, practice, and reality: a perspective on the molybdenum nitrogenase system

**DOI:** 10.1002/1873-3468.14534

**Published:** 2022-11-28

**Authors:** Stephanie D. Threatt, Douglas C. Rees

**Affiliations:** ^1^ Division of Chemistry and Chemical Engineering, Howard Hughes Medical Institute California Institute of Technology Pasadena CA USA

## Abstract

Nitrogenase is the sole enzyme responsible for the ATP‐dependent conversion of atmospheric dinitrogen into the bioavailable form of ammonia (NH_3_), making this protein essential for the maintenance of the nitrogen cycle and thus life itself. Despite the widespread use of the Haber–Bosch process to industrially produce NH_3_, biological nitrogen fixation still accounts for half of the bioavailable nitrogen on Earth. An important feature of nitrogenase is that it operates under physiological conditions, where the equilibrium strongly favours ammonia production. This biological, multielectron reduction is a complex catalytic reaction that has perplexed scientists for decades. In this review, we explore the current understanding of the molybdenum nitrogenase system based on experimental and computational research, as well as the limitations of the crystallographic, spectroscopic, and computational techniques employed. Finally, essential outstanding questions regarding the nitrogenase system will be highlighted alongside suggestions for future experimental and computational work to elucidate this essential yet elusive process.

## Graphical abstract

Nitrogenase performs a complex, multielectron reduction to convert dinitrogen into ammonia; this intricate catalytic reaction still perplexes scientists. In this review, we discuss the current understanding of the molybdenum nitrogenase system, limitations of contemporary approaches, and future directions for experimental and computational studies.

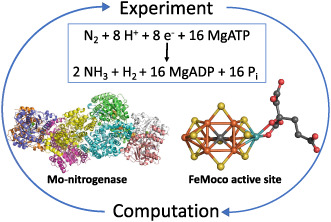

## Abbreviations

**ATP**, adenosine triphosphate

**DF**, density functional

**EPR**, electron paramagnetic resonance

**Fe protein**, iron protein

**FeMoCo**, iron molybdenum cofactor

**MoFe protein**, molybdenum iron protein

**N**_**2**_, dinitrogen

**N_2_ase**, nitrogenase

**NH**_**3**_, ammonia

Conversion of dinitrogen (N_2_) to the fixed form of ammonia (NH_3_) is essential for life, as fixed nitrogen is a key component of many biological molecules [1, 2]. Inert N_2_ is abundant, as it represents ~ 80% of Earth's atmosphere; however, this N–N triple bond represents one of the strongest bonds in nature [3] and often requires complex transition metal systems to promote nitrogen reduction. Namely, the industrial Haber–Bosch process and biological N_2_ fixation by nitrogenase (N_2_ase), which both feature multiple Fe centres, are responsible for generating nearly all of the bioavailable nitrogen on our planet. While the Haber–Bosch process represents a significant industrial advancement that has supported an increase of ~ 6 billion people in the global population over the past century, it requires high temperatures and pressures for catalysis and currently consumes approximately 2% of the world's annual energy production, while accounting for 1.4% of global carbon dioxide (CO_2_) emissions [4, 5, 6, 7]. By contrast, the biological process naturally operates under physiological conditions, where the equilibrium strongly favours ammonia production. Given the efficiency with which nitrogenase performs the catalytic conversion of N_2_ to NH_3_, there has been considerable research focused towards understanding the catalytic strategy employed for biological nitrogen reduction.

Biological nitrogen fixation is catalysed by the enzyme nitrogenase that naturally exists as three isozymes: Mo‐nitrogenase, V‐nitrogenase, and Fe‐nitrogenase, which contain different coordinating metals within their active site metallocofactors (molybdenum, vanadium, and iron, respectively) [8, 9, 10, 11, 12, 13, 14]. Most studies have focused on the molybdenum‐variant of nitrogenase (Mo‐N_2_ase), which has been well‐characterized and displays the greatest catalytic activity. The stoichiometry of the Mo‐N_2_ase reduction reaction is typically represented as follows:

$$N_{2}+8\;H^{+}+8\;e^{-}+16\;\text{MgATP}\to 2\;{\mathrm{NH}}_{3}+H_{2}+16\;\text{MgADP}+16\;P_{i},$$

with obligatory hydrogen (H_2_) evolution accompanying N_2_ reduction. While nitrogenase utilizes ecologically friendly energy sources as reducing equivalents, the Mo‐N_2_ase requirement for 16 MgATP molecules highlights that both the biological and industrial processes are energy consumptive. In contrast to the biological process that operates under ambient conditions, Haber–Bosch plants require high pressures between 200 and 400 atm and temperatures of 500 °C alongside notable methane consumption to source hydrogen, making the industrial process very energy intensive, as well as a potent source of greenhouse gas emissions [5]. Given the need for more efficient and sustainable strategies for large‐scale NH_3_ production, there have been decades of research dedicated to understanding the N_2_ase system. Studies of the Mo‐N_2_ase, V‐N_2_ase, and Fe‐N_2_ase systems have revealed structural and electronic similarities for all isoforms that support a universal, eight‐electron mechanism for N_2_ reduction by these N_2_ases [9, 11, 12, 15]; however, slight differences in the cofactor and surrounding protein scaffold between isoforms have provided unique insights about the general reduction mechanism. Importantly, many mechanistic details of the catalytic strategy employed by nitrogenase to successfully lower the activation barrier for N_2_ reduction relative to the industrial process remain enigmatic [16]; however, further understanding of the biological nitrogen reduction mechanism is essential for the development of more efficient catalysts for ammonia production that operate under mild reaction conditions while also being more sustainable.

This review will focus mainly on experimental characterizations of the Mo‐N_2_ase system, which have been aided by advances in spectroscopy and crystallography, as well as recent computational studies that have provided further insights into the role of various active site features and produced hypotheses for potential catalytic mechanisms (note, a 2020 review by Jasniewski et al. covers studies of the Fe‐ and V‐N_2_ase systems [11]). The limitations of both experimental and computational investigations of nitrogenase will then be described, followed by outstanding mechanistic questions. Lastly, recent developments with the potential to further elucidate this complex and essential catalytic process will be discussed.

## Experimental insights into biological nitrogen fixation

**Fig. 1:**
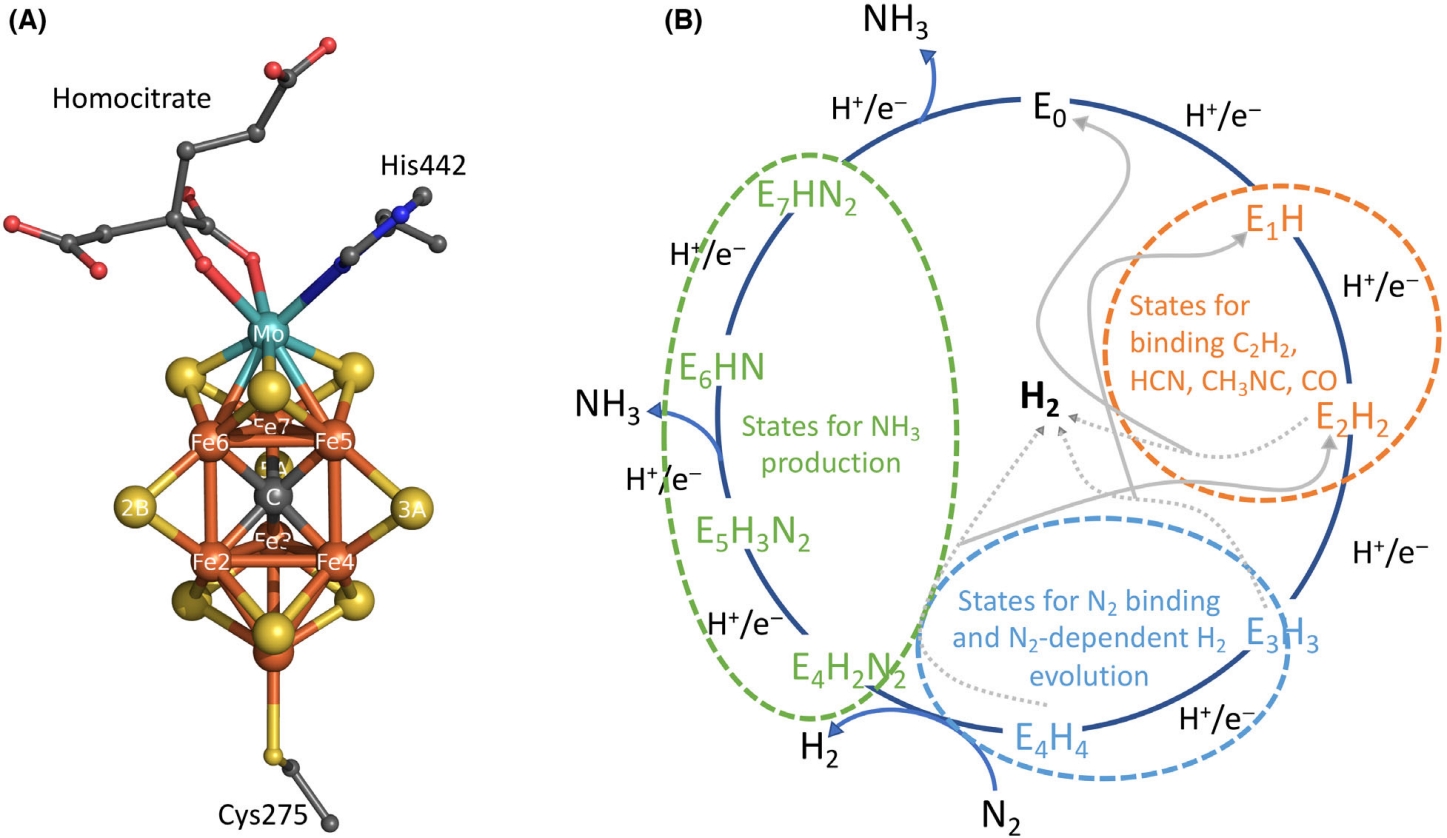
FeMoCo active site structure and Thorneley‐Lowe kinetic model for nitrogenase. (A) Structure of the FeMoCo active site and coordinating amino acids, Cys α275 and His α442, depicted in the E_0_ ground state. Numbering of atoms and residues is based on PDB deposit 3U7Q, and sulfur is shown in yellow, iron in orange, carbon in grey, oxygen in red, nitrogen in blue and molybdenum in cyan. (B) Schematic representation of the Thorneley‐Lowe model that describes the sequential eight‐electron and hydrogen transfer processes (E_0_–E_7_) postulated to result in ammonia (NH_3_) production catalysed by N_2_ase. Importantly, reduction of the cluster to the E_3_ or E_4_ state is necessary for N_2_ substrate binding to FeMoCo (circled in blue); however, other substrates have been found to bind the E_1_ and E_2_ states (circled in orange). H_2_ evolution is hypothesized to occur in states E_2_–E_4_. H_2_ evolution at the E_4_ state occurs through a reductive elimination reaction accompanying the binding of N_2_ to the metallocluster.

The well‐studied Mo‐N_2_ase system [8, 9, 10, 12] consists of two‐component metalloproteins that together catalyse the ATP‐dependent conversion of N_2_ to NH_3_: the molybdenum iron (MoFe) protein and the iron (Fe) protein. MoFe protein is an α_2_β_2_ tetramer that contains two metalloclusters, specifically an [8Fe:7S] P cluster and a [7Fe:9S:C:Mo]‐*R*‐homocitrate FeMo‐cofactor (FeMoCo) cluster (Fig. 1A). Importantly, FeMoCo operates as the Mo‐N_2_ase active site where substrates bind and undergo reduction. Fe protein is a homodimeric P‐loop ATPase with a bridging [4Fe:4S] cluster and two nucleotide‐binding sites sandwiched between the two subunits at the dimer interface. Fe protein is the obligate reductase for the N_2_ase system, and ATP hydrolysis by the Fe protein is coupled to electron transfer to the MoFe protein metalloclusters ([4Fe:4S] cluster → P cluster → FeMoCo) and ultimately substrate reduction. Mo‐N_2_ase must undergo reduction of the FeMoCo active site for initial binding of substrates to the cofactor to occur. Notably, various substrates can be reduced by N_2_ase, including N_2_, C_2_H_2_, CN^−^, $N_{3}^{-}$, and SCN^−^. Unexpectedly, N_2_ase can also catalyse C–C bond formation during the reduction of certain substrates, including CH_3_NC and CO [17, 18]; Einsle et al. have proposed a general mechanism for achieving C–C coupling [19]. Importantly, catalysis involves multiple sequential electron transfer steps that require the two‐component proteins to form a transient complex, where electron transfer is coupled to ATP hydrolysis.

Work by Burris and coworkers on *Azotobacter vinelandii* purified Mo‐N_2_ase established that a central feature of the enzyme's kinetic mechanism involves dissociation of the Fe protein‐MoFe protein complex [20]. They observed a lag phase in the production of H_2_, and a quantitative assessment of the relationship between the length of the lag phase and duration of turnover led to the hypothesis that dissociation of the N_2_ase protein complex is a necessary feature of biological nitrogen fixation. Their analysis of the electron paramagnetic resonance (EPR) signals from the active site of N_2_ase during turnover indicated that each cycle of complex formation and dissociation is associated with electron transfer from the Fe protein to the MoFe protein active site [21] and that ATP hydrolysis is coupled to these electron transfer steps. To reset for the next cycle, the Fe protein FeS cluster must get reduced, typically by electron transfer from either a flavodoxin or ferredoxin, and MgADP must be exchanged with MgATP.

In the 1970s, Lowe and Thorneley utilized *Klebsiella pneumoniae* Mo‐N_2_ase to generate a thorough kinetic mechanism for N_2_ reduction that is coupled with H_2_ evolution [22]. This model was inspired by Joseph Chatt's framework for the reduction of N_2_ proceeding through a sequence of electron–proton transfers in which N_2_ binding is associated the with displacement of H_2_ from the catalytic centre [23, 24]. The Thorneley‐Lowe model (summarized in Fig. 1B) postulates that various reduced states of the MoFe protein (E_*n*_) are generated throughout the N_2_ fixation cycle, where *n* refers to the number of electron transfer steps from reduced Fe protein, relative to the as‐isolated state (E_0_) of the MoFe protein. It was proposed that eight electron transfer cycles are necessary for the production of two equivalents of NH_3_ from N_2_, coupled to the evolution of one H_2_. Notably, N_2_ does not bind to the as‐isolated E_0_ form of FeMoCo (the state that has been most extensively characterized crystallographically). Instead, three or four proton and electron transfers to the FeMoCo cluster (generating the E_3_/E_4_ states) were found to be required before nitrogen substrate binding can occur. Hydrogen evolution occurs from FeMoCo states E_2_–E_4_ and hence can take place in the absence of N_2_ reduction. Other substrates, such as acetylene, cyanide, and isocyanides, have been found to bind to more oxidized forms of the MoFe‐protein compared to states associated with either hydrogen evolution or N_2_ binding [17, 25, 26].

Following the binding of N_2_ through a reductive elimination reaction associated with H_2_ evolution [27, 28], a series of alternating proton and electron transfers would result in the production of two equivalents of NH_3_. NH_3_ is proposed to be released from some combination of E_5_, E_6_, and/or E_7_ states. The rate‐determining step for the sequential transition between E_*n*_ states (*k* ∼ 6.4 s^−1^ at 23 °C for the *K. pneumoniae* system) was originally assigned as the dissociation of the Fe protein‐MoFe protein complex [29], but it is now associated with phosphate dissociation from Fe protein [30]. By utilizing the physiological reductant flavodoxin protein in the hydroquinone state, Yang et al. were able to evaluate rate constants of each key step in the N_2_ase reduction cycle and establish that phosphate dissociation from Fe protein after ATP hydrolysis is the rate limiting step. Additionally, this work indicated that the Fe protein only transfers a single electron to MoFe protein per ATP‐hydrolysis cycle. Furthermore, the same Fe protein binding interface was proposed to be used for MoFe protein and flavodoxin complexation [30].

Despite the ubiquity of the Thorneley‐Lowe scheme in providing the kinetic framework for N_2_ase, it is rarely used to quantitatively model experimental systems [16]. There are several reasons for this; most current experimental work employs *A. vinelandii* Mo‐N_2_ase assayed at 30 °C, while Thorneley and Lowe utilized the *K. pneumoniae* Mo‐N_2_ase at 23 °C. Independent attempts to quantitatively reproduce the reported kinetic fits were unsuccessful [31] and rapid freeze‐quench EPR studies resulted in reassignment of what should have been the E_3_ state based on kinetic modelling to the E_2_ state [32, 33]. Furthermore, FeMoCo states E_1_ through E_7_ were not directly detected and their existence was only inferred by Thorneley and Lowe; however, more recent spectroscopic experiments have observed E_1_, E_2_, E_4_, E_7_, and E_8_ MoFe reduced states [9]. A modified version of the Thorneley‐Lowe scheme has been developed by Hoffman and colleagues [9] that adds an E_8_ state and only focuses on E_4_ as competent for N_2_ binding [33]. Additionally, a steady‐state kinetic model for the *A. vinelandii* Mo‐N_2_ase has been recently described by Seefeldt and coworkers [14]. Taken together, these considerations indicate that the Thorneley‐Lowe model should be treated as a working hypothesis that will continue to evolve as experimental knowledge is amassed.

**Fig. 2:**
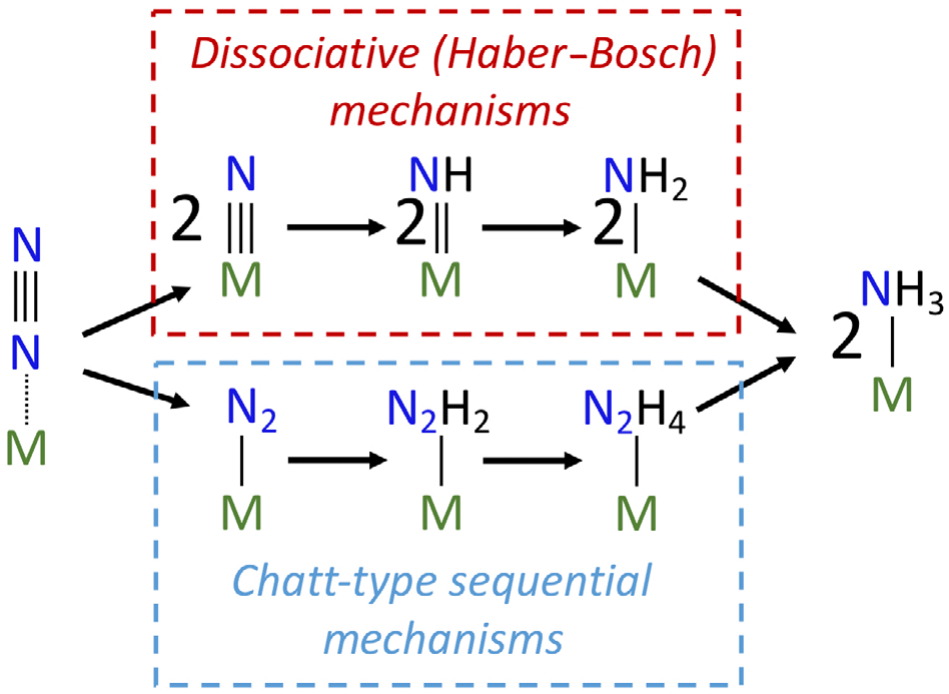
Possible mechanisms for N_2_ reduction to NH_3_. (Top) Dissociative mechanism typically associated with the Haber–Bosch process in which the N–N triple bond is cleaved during an initial step of the mechanism. (Bottom) Chatt‐type sequential mechanisms can proceed through distal, alternating, and hybrid pathways in which the N–N triple bond is progressively weakened *via* sequential hydrogenation (depicted using addition of pairs of H for simplicity). Variations include an initial reduction by multiple electrons, but without complete cleavage of the N–N triple bond [34], or reduction of N_2_ by a reaction other than hydrogenation [118].

Experimental insights are essential for framing hypotheses about the N_2_ase reduction mechanism, and all current work postulates that the biological conversion of N_2_ to NH_3_ occurs through sequential N–H functionalization as opposed to initial N–N triple bond cleavage as seen within dissociative mechanisms, such as the Haber–Bosch process or through a multi‐electron transfer step [34] (Fig. 2). Incremental weakening of the N–N bond through a sequence of coupled electron and proton transfers could proceed through distal, alternating, or hybrid reduction mechanisms [35, 36]. The distal mechanism would involve one N of N_2_ undergoing the first three hydrogenation steps to form and evolve NH_3_, and the remaining nitrido‐N is then hydrogenated three more times to produce the second NH_3_. Conversely, the alternating pathway could involve alternating hydrogenations of N_2_ where hydrogen transfers result in the stepwise formation of diazene (HNNH), hydrazine (H_2_NNH_2_), and then sequential production of NH_3_. Finally, a potential hybrid mechanism could involve a combination of hydrogenation steps from the described distal and alternating mechanisms. The main cited evidence for partially reduced NN intermediates is based on acid and alkali quenching experiments of N_2_ase under N_2_ turnover conditions yielding hydrazine [8, 37]; however, this is not a conclusive demonstration that hydrazine is an on‐path N_2_ase intermediate, as hydrazine is known to be generated during acid quenching studies of well‐characterized, synthetic N_2_ binding complexes [38].

Biochemical and spectroscopic studies continue to focus on evaluating the properties of various N_2_ase intermediates that are proposed to form during the Mo‐N_2_ase reduction mechanism. In particular, the “2N2H” intermediate has been assigned to the E_4_ state and proposed to correspond to a dinitrogen dihydride or diazene‐level intermediate [39]. Furthermore, a second E_4_ state that contains two hydrides, but no bound N substrate, has been characterized using freeze‐quenched EPR studies; this resulted in a hypothesis that reductive elimination of H_2_ is necessary for the generation of sufficiently reduced Fe centres for N_2_ binding, ultimately resulting in an E_4_ (2N2H) species. Importantly, E_5_ and E_6_ level intermediates that represent the subsequent steps in the reduction mechanism have yet to be detected.

X‐ray crystallographic studies have provided detailed structures of the N_2_ase component proteins, including the constituent metallocofactors, as well as more recent crystal structures of ligand‐bound forms of N_2_ase enzymes that have provided crucial mechanistic insights [10]. The first ligand‐bound Mo‐N_2_ase structure revealed that carbon monoxide (CO), a known inhibitor of N_2_ase substrates, becomes ligated between Fe2 and Fe6 atoms of FeMoCo in a μ_2_‐fashion replacing the S2B belt sulfur and resulting in hydrogen bonding between the O atom of the CO ligand and the imidazole of His195 [40]. Significantly, this region was implicated in substrate binding by Dean and colleagues based on the isolation and characterization of acetylene‐resistant mutants [41]. This work further builds upon infrared (IR), EPR, and electron‐nuclear double resonance (ENDOR) studies of Mo‐N_2_ase that had previously reported evidence of CO binding to a FeMoCo metal centre when subjected to various pressures of CO under turnover conditions [42, 43, 44, 45, 46, 47, 48, 49]. It is important to recognize, however, that crystallographic characterization of this species required a time scale of hours, which is much longer than the onset of inhibition (< 100 ms) and the time associated with appearance of the characteristic EPR signals of the CO inhibited state (~ 4 s) [42]. Interestingly, spectroscopic studies of CO‐inhibited MoFe protein in which the interstitial carbide is ^13^C enriched revealed that the process for CO incorporation at the belt sulfur positions does not involve major rearrangements of the CFe_6_ core [50]. Subsequent studies have confirmed the importance of the S2B site in ligand binding in both Mo‐N_2_ase [51] and V‐N_2_ase systems [19, 52, 53]. In particular, V‐N_2_ase studies have shown FeVCo is able to bind CO at the 2B position in the resting state. Interestingly, FeVCo does not require cluster reduction before ligand binding as observed with FeMoCo, likely due to a lower average valency of the Fe centres in FeVCo (V^3+^3Fe^3+^4Fe^2+^) compared to FeMoCo (Mo^3+^4Fe^3+^3Fe^2+^) [54, 55]. Taken together, these experiments highlight the privileged role in the binding of exogenous ligands played by Fe2 and Fe6 in the trigonal prism of the FeMoCo.

Additional structural studies with selenocyanate (SeCN^−^) have unambiguously demonstrated that exogenous ligands can become incorporated into the belt positions, especially S2B. In particular, when N_2_ase is incubated with SeCN^−^ under turnover conditions, Se is initially incorporated primarily, but not exclusively, at the S2B position. When the Se2B‐labeled MoFe protein is subsequently subjected to turnover in the presence of acetylene, Se migration to the S5A and S3A belt sulfur positions is observed [56]. Alternatively, when the Se2B‐labeled protein and active site are treated with CO, Se migrates to the S5A and S3A positions as opposed to being removed from the cofactor; this result suggests a process in which the cofactor either rearranges to exchange the belt positions or an overall rotation of the cofactor within the active site takes place. While the exact mechanism for FeMoCo belt sulfur rearrangements remains enigmatic, these SeCN^−^ and CO N_2_ase ligand studies generally establish that Fe–S bonds are labile and structural rearrangements can occur within the FeMoCo active site during turnover [56, 57]. Further work is needed, however, to establish whether the belt sulfur displacement observed in these ligand binding studies represents an “on‐path” state in the N_2_ reduction mechanism or is representative of “off‐path” species formed under these experimental conditions.

## Limitations of N_2_ase experimental studies

While experimental studies of N_2_ase have undoubtedly provided crucial insights, it is important to recognize that many details of the reduction mechanism remain elusive. Indeed, the combination of the small size of the dinitrogen substrate, the inability of the as‐isolated form of FeMoCo to bind N_2_, and the impressive complexity of the active site have hindered the deciphering of an atomic‐level understanding of the biological mechanism for N_2_ reduction. Furthermore, at steady state, a mixture of states (E_0_–E_7_) will be present, making concrete interpretations of biochemical and spectroscopic data very challenging. The presence of the P‐cluster ([8Fe:7S]) and 4Fe4S clusters of the Fe protein further complicate the analysis of spectroscopic data, as does the prediction that approximately half of the Mo‐N_2_ase N_2_ substrate intermediates will be even‐electron and thus “dark” when analysed with spin resonance techniques. Signal relaxation‐broadening above 25 K further confounds spectroscopic analysis of vital Fe–N species [58]. Notably, studies of dithionite‐reduced FeFeCo revealed that Fe‐N_2_ase has an integer spin ground state likely caused by antiferromagnetic coupling between the cofactor Fe atoms that results in odd‐electron states (E_1_, E_3_, E_5_, E_7_) rather than even‐electron states (E_0_, E_2_, E_4_, E_6_) being paramagnetic and spin‐resonance technique active [59]. Resultingly, spin resonance studies that couple data from the MoFe and FeFe proteins should allow for all relevant reduced states of the N_2_ase cofactor to be fully characterized. Additionally, it has been well‐documented that the nitrogenase proteins exhibit considerable oxygen sensitivity and thus must be manipulated within anaerobic environments. Most N_2_ase protein samples are accordingly supplemented with dithionite (Na_2_S_2_O_4_) in order to prevent O_2_ contamination [60, 61]. Dithionite (DT) is known to gradually decompose within aqueous solutions to a variety of S‐containing species, including sulphite (${\mathrm{SO}}_{3}^{2-}$), sulfate (${\mathrm{SO}}_{4}^{2-}$), and sulphide (S^2−^) [60, 62], and these decomposition products have the potential to react with the FeMoCo under turnover conditions, which could prevent detection and isolation of activated, reduced forms of the cofactor.

Together, experimental studies of Mo‐N_2_ase indicate that a complex sequence of proton and electron transfer steps associated with hydride formation, and likely coupled with cofactor rearrangement, govern activation of N_2_; however, few details are known about the intermediate FeMoCo turnover states. Given the small sizes of N_2_ase substrates and the existence of multiple conformational states under turnover conditions, approaches are limited for detailed experimental characterization of substrate reduction intermediates. Thus, computational techniques serve a crucial role in elucidating the specific mechanistic steps of biological nitrogen fixation. Importantly, theoretical studies have previously clarified mechanistic steps of redox‐active enzymes involving multiple electron and proton transfer steps, such as photosystem II [63, 64, 65]. Given the limitations of current experiments to provide detailed characterization of FeMoCo reduction intermediates, their integration with complementary computational studies is essential to further illuminate the complicated process of N_2_ase reduction.

## Computational insights into nitrogenase reduction of N_2_

As we have discussed, experimental investigations of N_2_ase serve as the basis for framing the chemical mechanism of biological nitrogen fixation. Despite substantial experimental efforts towards elucidating specific details of this system, including N_2_ binding to the cofactor concomitant with release of H_2_, N–N bond cleavage and N–H bond formation *via* iterative proton and electron transfer steps, and release and transport of NH_3_ to the protein surface, many features of the N_2_ fixation mechanism require further validation. As such, computational studies of the N_2_ase system are essential for simulating proposed reactions and pathways to illuminate the atomic details of the molecular mechanism of biological nitrogen fixation.

The accumulation of protons and electrons on FeMoCo is key to priming the active site for substrate binding and results in the formation of a variety of E_*n*_H_*n*_ species. One key advantage of computational studies is the ability to simulate and evaluate a single, pure intermediate species and thereby assess its mechanistic feasibility and relevance. Various quantum mechanics (QM) and quantum mechanics/molecular mechanics (QM/MM) model studies have explored these E_*n*_H_*n*_ structures to determine the likely sites for proton and hydride accumulation prior to N_2_ binding. Ryde and Cao have established that the resting state (E_0_) is deprotonated and the E_1_H_1_ state has one H on belt sulfur S2B, which affords > ~ 20 kJ·mol^−1^ energy stabilization [66]. Studies of substrate‐free E_2_H_2_, E_3_H_3_, and E_4_H_4_ states in particular have revealed the importance of functional choice in density functional (DF) calculations of FeMoCo. The pure TPPS functional often favours structures with Fe–H bonds, whereas hybrid functionals like B3LYP tend to significantly favour formation of C–H bonds by the interstitial carbon [66]. Ultimately, given the large number of possible combinations of geometrical and electronic structures of FeMoCo, a conclusive understanding of the electronic and structural states that correspond to E_1_H_1_ through E_4_H_4_ have yet to be determined; however, some consistent trends are evident in the computational findings. For instance, formation of S2B‐H is energetically favourable, when S3B is protonated it consistently loses a bond to Fe not Mo, and calculations where the electronic state is defined initially often undergo changes during optimization to a different electronic state or related geometrical structure [67].

An essential step of each N_2_ reduction cycle involves an N_2_ molecule navigating from the protein surface to the FeMoCo active site. Computational simulations and examination of the crystallographic binding sites of small molecules, such as xenon, to the MoFe‐protein have revealed multiple potential substrate channels, including a hydrophobic path that leads to the Fe2 position of FeMoCo [68, 69, 70, 71, 72, 73, 74]. Free energy analyses of N_2_ moving from the protein surface to FeMoCo through this channel have revealed extremely low activation‐free energy barriers (~ 12 kJ·mol^−1^), further supporting feasibility of this path as an N_2_ ingress channel [68, 70]. Fe2 has been implicated as an important site for N_2_ binding based on multiple computational studies; details of the potential roles of Fe2 in the N_2_ase mechanism are discussed in greater detail in the full mechanism section below [75]. Relatedly, DF studies focused on the role of the interstitial carbide (C^c^) have revealed hydrogenation, and the binding of substrates and intermediates results in coordinative allosterism within FeMoCo. For example, DF studies have shown that N_2_ binding to Fe2 results in weakening and elongation of the Fe2–C^c^ bond and compensatory strengthening and shortening of the Fe6–C^c^ bond; satisfying a proposed five bond optimum bonding capacity of the C^c^ to Fe atoms in FeMoCo [67, 76]. Note that studies where the interstitial carbide of FeMoCo is ^13^C labelled indicate the CFe_6_ cluster core does not experience significant rearrangements within the states that have been examined spectroscopically [50].

The physiological process of N_2_ reduction requires eight proton transfer steps to produce 2NH_3_ and 1H_2_. DF calculations have explored mechanisms for serial supply of protons to FeMoCo, and His195, the closest side chain to the FeMoCo cluster, is anticipated to have a p*K*_a_ in the physiological range, that would likely allow for at least one proton transfer step [77, 78]. It has been postulated that the relative high acidity of the protonated imidazole may indicate hydrogen transfer from His195 represents the first and most difficult reduction step in the N_2_ fixation process (NNH formation); however, simulations have hypothesized that continual relay of protons from His195 may require a complete imidazole flip, which has a higher energy barrier [78]. Consequently, other proton supply mechanisms have been explored, and a water chain that connects the protein surface to S3B has been identified and analysed. This single chain of eight hydrogen‐bonding water molecules has been strictly conserved across all reported crystal structures of MoFe protein [79]. It is proposed that this water chain acts as a proton wire that allows for hydrogens to accumulate on S3B *via* the standard Grotthuss mechanism [80, 81]. A 269‐atom DF model that included all pertinent residues near the water chain revealed that there were low potential energy barriers (< 30 kJ·mol^−1^) for proton movement across the wire; this process also included the participation of carboxylate groups of the homocitrate. Notably, once S3B‐H is formed, the H atom can move to different sites on S3B, which allows for hydrogen transfer to other sites within FeMoCo. DF studies of the various S3B‐H configurations revealed that the potential energy surface barriers for H bond movement are small, and one key simulated H atom transfer pathway involved hydrogen movement from S3B to Fe6 and finally to S2B and Fe2, which could allow for hydrogenation of substrates and intermediates [80, 82, 83]. Ultimately, computational studies of hydrogen accumulation processes for FeMoCo have clarified our understanding of potential roles for His195, *R*‐homocitrate, and the conserved water chain adjacent to FeMoCo as crucial towards the formation of E_*n*_H_*n*_N_*x*_ intermediates.

**Fig. 3:**
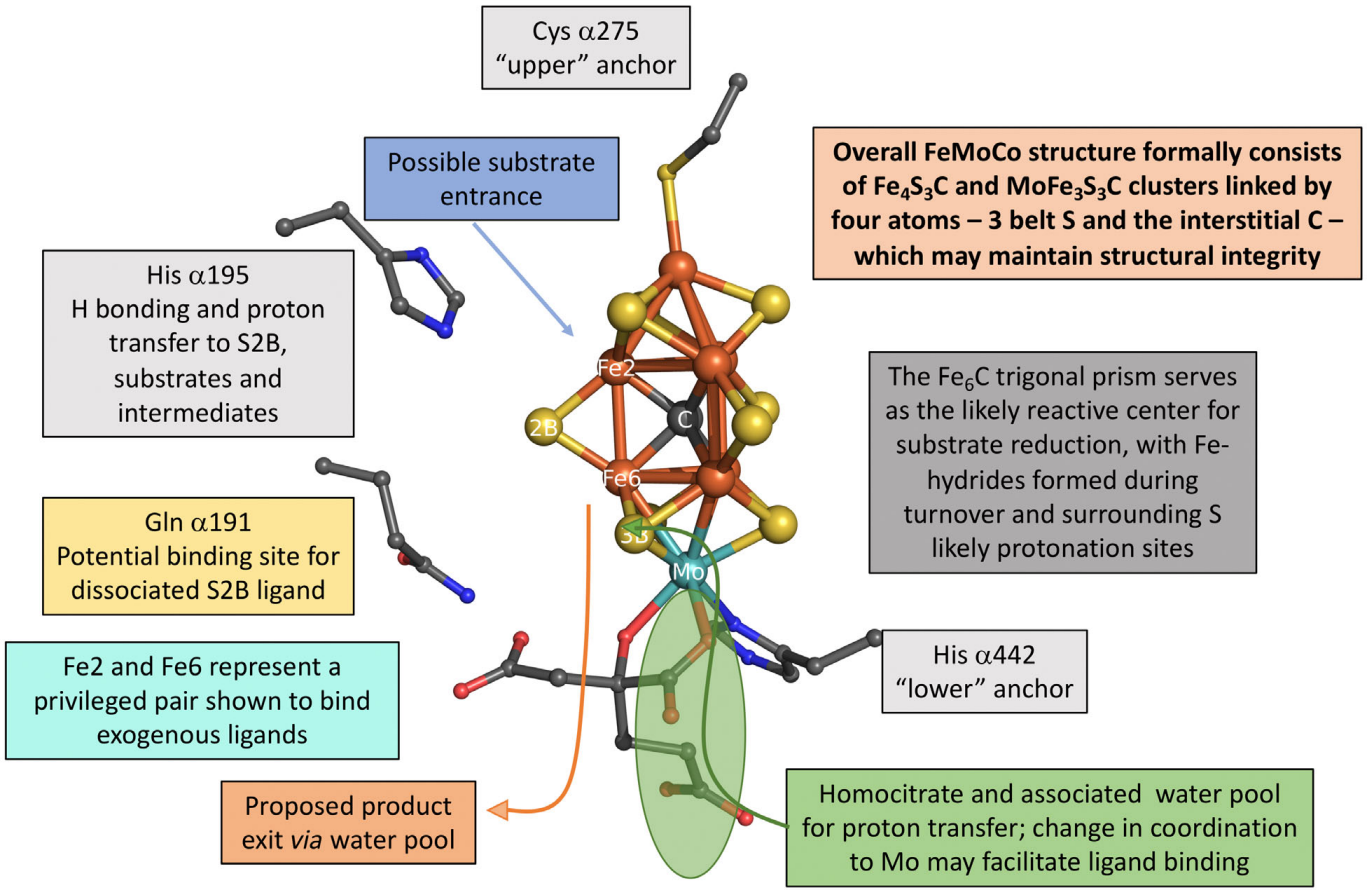
Summary of the mechanistic roles of structural components within the nitrogenase active site. Proposed mechanistic functions and roles for key features of the FeMoCo active site, including His α195, Gln α191, Cys α275, His α442, FeMoCo water pools, homocitrate, interstitial carbide, as well as the overall cofactor structure are outlined. Figure adapted from Dance 2020 review [79].

Finally, computational studies have explored the NH_3_ exit pathway and identified a homocitrate‐associated pool of water molecules that are believed to assist in the egress of the ammonia product [74]. Analysis of the protein tertiary structure and probable dynamic fluctuations associated with NH_3_ transport to the protein surface have been modelled. The relatively high B factors of this region support conformational flexibility potentially associated with the N_2_ase mechanism and NH_3_ product egress. Insights regarding the FeMo‐cofactor structure and roles of various components of the active site are summarized in Fig. 3 (adapted from [83]).

## Comparing computationally proposed N_2_ reduction mechanisms

Dance [79] has reviewed a collection of computationally‐based N_2_ reduction mechanism proposals, including work by Blöchl, Kästner et al. [84, 85, 86, 87], Nørskov et al. [88, 89]. and Adamo et al. [90]. Here, we will discuss selected features of these mechanisms, including recently published updates. Work by Dance investigating the association between N_2_ binding and Fe–S bond lability revealed the importance of multi‐atom rearrangements. The DF calculations were based on experimentally derived structural features, including a core cluster ([Fe_7_MoS_9_C]) charge of −1, net E_0_ spin state of *S* = 3/2, atomic coordinates from the 1 Å resolution 3U7Q PDB X‐ray crystal structure (486‐atoms total), and mutagenesis and structural studies that implicate the Fe2‐Fe3‐Fe6‐Fe7 face as the catalytic reaction zone of FeMoCo [32, 91, 92, 93, 94]. Given that Fe2 is located near a probable channel for ingress diffusion of N_2_ and His195 can provide hydrogen bonding to a Fe2‐coordinated substrate, N_2_ binding to the Fe2 position was simulated. Initially, FeMoCo was modelled to have a hydrogen bridging Fe2 and Fe6 and an additional hydrogen binding to S2B, the belt sulfur that connects Fe2 and Fe6. Upon equilibration of N_2_ within the ingress channel (initial distance 3 Å), N_2_ was found to bind Fe2 end‐on with a very small energy barrier (12 kJ·mol^−1^ when *S* = 1/2 or 16 kJ·mol^−1^ *S* = 3/2), accompanied by dissociation of S2B‐H from Fe2 [75]. Notably, more extensive rearrangements that would lead to internal scrambling of the belt positions were found to have a high barrier and hence would be unlikely to occur [95]. More recent work by Dance has explored the possibility that the N_2_ molecule that initially binds Fe2 *via* the substrate channel may not be reduced, but instead could play a promotional role in expanding the reaction zone [96]. This mechanism proposes that a second N_2_ enters *via* the ingress channel and binds to Fe6, where it is primed for intramolecular hydrogen transfer from atoms originating on S3B, culminating in reduction from N_2_ to NH_3_ [96].

A critical feature in the N_2_ase mechanism of Siegbahn concerns the role of the interstitial carbide. By analysing models with 160–270 atoms using the B3LYP hybrid functional, Siegbahn has identified a mechanism in which C^c^ becomes hydrogenated to a methyl group that shifts to the periphery of the cluster core while bound to Fe6 (*exo*‐Fe6) [77, 97, 98]. Subsequently, N_2_ substrate binding and hydrogenation are proposed to proceed within the now empty central region. Importantly, formation of CH_3_ is categorized as an “activation phase” that is equivalent to the E_0_ state. The mechanism then proceeds *via* hydrogenation steps that mimic the Thorneley‐Lowe model, and E_4_‐H_2_/E_4_‐N_2_ exchange is included. The distinctive structural features of this Siegbahn hydrogenation cycle include the role of CH_3_ bound *exo* to Fe6, unhooking of S2B‐H from Fe6 during the hydrogenation and rehooking afterwards, as well as generation of five‐coordinate Mo due to partial dissociation of the homocitrate alcohol group [79]. Wei and Siegbahn recently reported an additional analysis of the initial four reduction activation steps (generating E_0_) that resulted in loss of a sulfide (H_2_S3A) as opposed to protonation of C^c^ to a methyl group. This builds upon experimental studies that showed the inhibitor CO can replace S2B [40]. Simulation of N_2_ binding revealed that the nitrogen substrate binds too weakly to force loss of a sulfide; however, CO binds significantly more strongly to FeMoCo and successfully displaces the sulfide in an almost thermoneutral step based on these DF studies. Overall, the four activation steps were found to result in a very weakly bound sulfide that could be released in a significantly exergonic step (−50 kJ·mol^−1^). This study found that the barrier for protonation of the carbide beyond CH was much higher than sulfur protonation. Additionally, after sulfide loss, the catalysis cycle was modelled to commence *via* N_2_ binding to Fe4, one of the most reduced Fe atoms in this system. Notably, sulfide loss was also associated with hydrides preferring a bridging position, which had a stabilizing effect on the overall cluster [99]. It should be noted that an *exo* Fe‐CH_3_ group would likely be hydrolytically unstable and a detailed mechanism for regeneration of the E_0_ resting state has yet to be clearly outlined [79].

Ryde has explored the N_2_ase reduction mechanism using computational studies, with recent work focusing on the potential role of the μ_2_‐bridging S2B position in substrate binding and reduction [100, 101, 102]. In particular, a QM/MM approach (133 915 atoms) was paired with QM calculations (170–178 atoms), where two DF methods were employed: the non‐empirical, pure functional TPSS and the hybrid B3LYP functional, to explore the optimized energies and geometries of a wide range of NNX‐bound E_4_ structures. This full N_2_ase mechanism is proposed to begin with N_2_ binding in a “half‐bridging” mode within the empty S2B site. N_2_H_2_ is then produced using intramolecular hydrogen transfer from protons already present on the cluster with the first protonation requiring approximately 17 kJ·mol^−1^ of energy. Interestingly, Cao and Ryde suggest N_2_ hydrogenation most favourably proceeds from intermediate NNH_2_ bound end‐on to H_2_NNH in a side‐on bridging conformation (Fe2/Fe6). Formation of H_2_NNH_2_ (hydrazine) was then reported to be the most stable subsequent structure, and the next reduction step facilitated facile cleavage of the NN bond resulting in NH_3_ bound solely to Fe2 and ${\mathrm{NH}}_{2}^{-}$ stabilized by a bridging conformation between Fe2 and Fe6. The final mechanistic step involves NH_3_ dissociation and protonation of ${\mathrm{NH}}_{2}^{-}$ to NH_3_, but only after S2B rebinds to the cluster. Overall, this work proposed a hybrid mechanism, in which E_5_‐E_7_ states favour an alternating mechanism (with the exception of NNH_2_ being reported as the most stable N_2_H_2_ state), while other steps in the mechanism suggest a distal approach, such as favourable formation of NNH_3_. This computational work builds upon experimental studies to suggest N_2_ could bind to an empty S2B site and coordination of nitrogen substrate between Fe2 and Fe6 atoms may facilitate the formation of two NH_3_ molecules *via* an energetically favourable mechanism.

## Limitations of N_2_ase computational studies

Computational studies avoid many of the limitations inherent with experiments and can provide novel insights into the unprecedented structure and coordination chemistry of FeMoCo. As detailed above, various theoretical analyses of the N_2_ase system have provided important insights ranging from identification of likely substrate ingress and egress channels to proposed detailed mechanisms for the entire reduction process; however, computational analyses are also not without limitations. Each of these studies involves constraining the parameters of the nitrogenase system based upon the focus of that particular study, such as deciding the number of atoms used (model size), choice of functional and basis set, defining the overall model charge, and choice of solvation dielectric constant. Defining the functional choice, particularly hybrid vs. pure, has been found to especially influence which FeMoCo structures and bonds are energetically and geometrically favoured with wide variations reported (> 200 kJ·mol^−1^) [103]. As such, this review has focused on key features of mechanisms that have been proposed through computational studies. Clearly, these features cannot all be correct, but they highlight aspects of the mechanism that can ultimately be validated through an iterative process of hypothesis revision coupled with continued advances in both computational and experimental studies.

## Outstanding questions and future studies

Advances in experimental and computational techniques have aided our understanding of several facets of biological nitrogen fixation. Given the complexity of the metallocluster active site and current inability to trap mechanistically relevant intermediates, outstanding questions regarding the N_2_ase system include:

1. What are the mechanistically relevant intermediates for N_2_ fixation? Current models have singularly focused on a general mechanism where N_2_ reduction proceeds through a sequence of proton and electron transfers, but it must be noted that no definitive evidence for any on‐path, partially reduced NN intermediate has been presented.
2. Does the cofactor rearrange during turnover? In particular, how do the belt sulfurs exchange/interchange during turnover? Does the CFe_6_ core remain rigid throughout N_2_ reduction?
3. Does each cycle of reduction by the Fe protein result in the coupled transfer of one proton and one electron to the FeMoCo, as typically modeled?
4. What is the exact role of His195 within the substrate reduction mechanism? Can this residue supply multiple hydrogens to the cofactor?
5. Is the H_2_/N_2_ exchange step accompanied by hydrogenation of N_2_? Is this a unique reaction associated with N_2_ reduction, or do other substrates also display this property?
6. What are the roles of homocitrate in the substrate reduction mechanism?

These queries represent only a few of the unresolved details regarding this system, and future experiments will need to combine findings from Mo‐N_2_ase experimental and computational work, as well as evaluations of the Fe‐N_2_ase and V‐N_2_ase systems, in order to advance our knowledge of this process. There are a number of promising approaches to further illuminate steps in the N_2_ase mechanism. Experimentally, strategies for the site‐specific labelling of various atoms in the FeMo‐cofactor, including iron [104], molybdenum [105], carbon [50, 106], sulfurs (including selenium incorporation) [56, 57, 62], and homocitrate [107] will allow for transformative spectroscopic and structural studies that should yield insights into bonding interactions, rearrangements, and potentially allow for substrate coordination sites to be monitored. Another arena for important experimental advances is in the development of Fe protein‐independent systems that utilize a photo‐activated supply of electrons [108, 109] or electrocatalysis [110, 111] to reduce the MoFe protein; this would allow for the electron supply to be better controlled to more effectively generate homogenous samples in defined E_*n*_ states for analysis. The recent revolution in electron microscopy is opening up structural studies of solutions of nitrogenase in defined states [112, 113, 114], as well as under turnover conditions [114]. Computational analyses will benefit from advances that allow for more realistic treatment of complex metalloclusters [115, 116], as well as to incorporate more of the surrounding protein and account for the dynamics of the protein structure on the chemistry within the active site. Computational and experimental studies will both benefit from synthetic FeS clusters that mimic FeMoCo and support the binding [117] and particularly the reduction of N_2_ [118]. Ultimately, the integration of experimental and computational studies will be essential for the identification and analysis of relevant intermediate species that are central to the N_2_ase mechanism for biological nitrogen fixation.
